# Development and Application of a Slot-Blot Assay Using the Damage Sensing Protein Atl1 to Detect and Quantify *O*^6^-Alkylated Guanine Bases in DNA

**DOI:** 10.3390/toxics12090649

**Published:** 2024-09-04

**Authors:** Hanum Yaakub, Anthony Howell, Geoffrey P. Margison, Andrew C. Povey

**Affiliations:** 1 Epidemiology and Public Health Group, Division of Population Health, Health Services Research and Primary Care, School of Health Sciences, Faculty of Biology, Medicine and Health, University of Manchester, Manchester M13 9PL, UK; hanumyaakub@iium.edu.my (H.Y.); gmargison@manchester.ac.uk (G.P.M.); 2 Prevent Breast Cancer Centre, Wythenshawe Hospital Manchester Universities Foundation Trust, Wythenshawe, Manchester M23 9LT, UK; anthony.howell@manchester.ac.uk; 3 Manchester Breast Centre, The Christie NHS Foundation Trust, Wilmslow Road, Manchester M20 4GJ, UK; 4 Division of Cancer Sciences, School of Medical Sciences, Faculty of Biology, Medicine and Health, University of Manchester, Manchester Academic Health Science Centre, Manchester M13 9PL, UK

**Keywords:** *N*-nitroso compounds, *O*^6^-alkylguanines, Atl1, MGMT, slot-blot assay

## Abstract

Humans are unavoidably exposed to numerous different mutagenic DNA alkylating agents (AAs), but their role in the initiation of cancers is uncertain, in part due to difficulties in assessing human exposure. To address this, we have developed a screening method that measures promutagenic *O*^6^-alkylguanines (*O*^6^-AlkGs) in DNA and applied it to human DNA samples. The method exploits the ability of the *Schizosaccharomyces pombe* alkyltransferase-like protein (Atl1) to recognise and bind to a wide range of *O*^6^-AlkGs in DNA. We established an Atl1-based slot-blot (ASB) assay and validated it using calf thymus DNA alkylated in vitro with a range of alkylating agents and both calf thymus and human placental DNA methylated in vitro with temozolomide (TMZ). ASB signals were directly proportional to the levels of *O*^6^-meG in these controls. Pre-treatment of DNA with the DNA repair protein *O*^6^-methylguanine–DNA methyltransferase (MGMT) reduced binding of Atl1, confirming its specificity. In addition, MCF 10A cells were treated with 500 μM TMZ and the extracted DNA, analysed using the ASB, was found to contain 1.34 fmoles *O*^6^ -meG/μg DNA. Of six human breast tumour DNA samples assessed, five had detectable *O*^6^-AlkG levels (mean ± SD 1.24 ± 0.25 *O*^6^-meG equivalents/μg DNA. This study shows the potential usefulness of the ASB assay to detect and quantify total *O*^6^-AlkGs in human DNA samples.

## 1. Introduction

*N*-nitroso compounds (NOCs) have long been known as potent animal carcinogens [1,2] and some are known or suspected to be human carcinogens [3]. Human exposure via the diet [4,5] and other sources [6,7] is well-documented. Endogenous nitrosation of amines has also been implicated as a source of these agents [8,9,10]. However, due to this wide range of exogenous and endogenous exposures, establishing the contribution of NOCs to the human cancer burden is not straightforward.

NOCs undergo metabolic activation [11] and the products of this can alkylate cellular macromolecules including DNA [12]. Damage to DNA results in covalent modifications (“adducts”) at oxygen and nitrogen atoms of DNA bases and the oxygen atoms in phosphodiesters forming phosphotriesters [11,12,13]. The relative amounts of these adducts vary according to the chemical properties of the alkylating species [14]. For methylating agents, the predominant adduct formed is N7-methylguanine, whereas for higher alkylating agents (AAs), phosphotriesters can be the major lesions [14]. These lesions do not appear to contribute significantly to the adverse toxic, mutagenic and carcinogenic effects of NOC, whereas *O*^6^-alkylguanine (*O*^6^-AlkG) adducts, generally formed in relatively lower amounts, are biologically more potent [15,16]. In particular, *O*^6^-alkGs are considered highly mutagenic causing mainly GC to AT transitions during DNA replication [13,16,17]. Such mutations have been seen in human tumour DNA following treatment with the alkylating agent temozolomide (TMZ) [18] and a mutational signature consistent with alkylating agent exposure has been described in colorectal tumours [19].

A number of different *O*^6^-alkG adducts have been detected in human DNA including *O*^6^-methylG (*O*^6^-MeG), *O*^6^-ethylguanine, *O*^6^-(carboxymethyl) guanine (*O*^6^-CMG) [20,21,22]. *O*^6^-AlkGs in DNA can be repaired by *O*^6^-methylguanine DNA methyltransferase (MGMT) which stoichiometrically transfers the alkyl group to a cysteine (Cys) residue within the protein active site resulting in self-inactivation [23,24]. Thus, MGMT is a key factor in defending cells against AA-induced toxicity, mutagenicity and carcinogenicity [25]. We have recently exploited this mechanism to detect *O*^6^-AlkGs in human DNA using matrix-assisted laser desorption/ionisation–time-of-flight mass spectrometry (MALDI-ToF) methodology to analyse the MGMT active site tryptic peptide following in vitro incubation of recombinant MGMT with tumour and normal tissue DNA [26]. The incubation of human colorectal DNA with recombinant MGMT resulted, in addition to methylated and carboxymethylated MGMT active site peptides, in a putative hydroxyethylated modified active site peptide and other, as yet unidentified alkyl group modifications of mass between 144 and 240 [26]. Based on a number of studies of their coding properties in DNA, it seems reasonable to assume that any *O*^6^-AlkG is potentially mutagenic and hence carcinogenic. Therefore, a method that can determine all *O*^6^-AlkGs in a single assay could be a distinct advantage in terms of assessing their potential contribution to cancer.

In *Schizosaccharomyces pombe*, the alkyltransferase-like protein 1 (Atl1) strongly binds *O*^6^-AlkG adducts in DNA but unlike MGMT, which has a cysteine residue in the DNA binding pocket, Atl1 has a tryptophan and is unable to undertake alkyl group transfer [23]. This DNA-damage-sensing protein provides protection against the toxic effects of a number of alkylating agents [27], by enabling nucleotide excision repair to act on *O*^6^-AlkGs in DNA [28]. Given that Atl1 binds a wide range of *O*^6^-AlkGs in DNA [28], we have exploited it to develop an Atl1 slot-blot (ASB) assay that enables the direct quantification of total *O*^6^-AlkGs in human DNA samples.

## 2. Materials and Methods

### 2.1. Materials

Temozolomide (TMZ; catalogue # 2706) was purchased from Tocris, allylnitrosourea (ANU) and benzylnitrosourea (BZNU) were supplied by Cruachem; butylnitrosourea (BNU), ethylnitrosourea (ENU), potassium diazoacetate, propylnitrosourea (PNU) were all synthesised in house. All are direct acting alkylating agents and potential mutagens and carcinogens and should be handled with care in a well-ventilated fume hood using gloves and other PPE.

Atl1 (Uniprot Q9UTN9) was expressed as a maltose-binding protein (MBP) fusion protein from a pMAL-2c expression vector construct and affinity purified using amylose resin (New England Biolabs Inc., Ipswich, MA, USA) essentially as described previously for the *E. coli* protein [27]. MBP lacks the very highly conserved active site peptide domain that is most frequently PCHRV in MGMT or PWHRV in Atl1 [23] and binding to *O*^6^-AlkGs is not expected. MBP-Atl1 was conjugated to horseradish peroxidase (HRP; Abcam, Cambridge, UK) as described in the user manual. Human MGMT (Uniprot P16455) was also expressed as a hexahistidine (His) fusion protein from pQE30Xa (Qiagen, Manchester, UK) and purified by nickel affinity chromatography using complete His-Tag purification resin (Sigma-Aldrich, Poole, UK) [26]. Salmon testes (Catalogue number 262012) and *Micrococcus luteus* (D8259) DNA were also obtained from Sigma-Aldrich. DNA was extracted from human breast and the middle, edge and cord regions of the maternal side of villous placental tissue and MCF 10A human breast epithelial cells (a gift from Dr Jonathan Humphries; University of Manchester) using the Blood and Cell Culture DNA Kit (Qiagen, Manchester, UK).

### 2.2. Preparation of DNA Samples Containing a Variety of O^6^-AlkG Adducts

To assess the ability of the ASB to detect different *O*^6^-AlkGs, DNA samples containing different *O*^6^-AlkGs were prepared as follows: TMZ (2.3 mg) ENU (11 mg) PNU (11 mg) BNU (5.7 mg) ANU (5 mg) and BzNU (5 mg) were each dissolved in 50 μL DMSO. To each was added 500 μL calf thymus (CT) DNA (Sigma-Aldrich, Poole, UK) dissolved at 2 mg/mL in 100 mM Tris 25 mM EDTA pH 8. After incubation at 37 °C for 3 h, the DNA was precipitated by adding 50 μL 3 M NaAc and 1 mL ethanol, vortex mixed and stored overnight at −20 °C. The DNA precipitate was collected by centrifugation, washed with ethanol, air dried, dissolved in 500 μL TE, reprecipitated and washed and finally dissolved in 1 mL TE and stored at −20 °C. For reaction with KDA, potassium hydroxide (1.14 g) was dissolved in water (11.4 mL) and to this was added 1.06 mL of ethyl diazoacetate (Sigma-Aldrich, Poole, UK). The mixture was stirred at room temperature for 5 h and the resulting potassium diazoacetate (KDA) stored in aliquots at −20 °C without purification. To 500 μL CT DNA, dissolved in TE at 5 mg/mL, was added 5 uL of KDA (final concentration ~8 mM) and the mixture incubated at 37 °C overnight, then processed as above.

### 2.3. Production of CT DNA Standards of Known O^6^-MeG Levels

CT-DNA (Sigma-Aldrich, Poole, UK) and human placental DNA were incubated at 37 °C with different concentrations of TMZ (0.2–20 μg/mL [1.03–103 μM] and 0.375–1.5 μg/mL [1.9–7.7 μM] DMSO, respectively). *O*^6^ -MeG levels were determined using an MGMT-based competition assay which involves incubating known amounts of the TMZ-treated DNA with a fixed amount of MGMT under substrate-limiting conditions then incubating with excess [^3^H]-methylated DNA and determination of the residual amount of MGMT activity which then relates to the amount of *O*^6^-MeG in the unlabelled DNA [29]. To reflect the levels of *O*^6^-MeG previously reported in human DNA samples (e.g., [22]), the levels of *O*^6^-MeG in the standards ranged from 0.278 to 27.8 fmoles/μg DNA (0.167–16.7 × 10^10^ molecules/μg DNA) in CT DNA and 0.507 to 2.035 fmoles/μg (0.304–1.22 × 10^10^ molecules/μg DNA) in placental DNA.

### 2.4. ASB Assay

DNA concentrations were determined using a NanoDrop spectrophotometer (Thermo Fisher Scientific, Altrincham, Cheshire, UK) and adjusted to 10 μg/mL in 10 mM Tris-HCl, 1 mM EDTA (TE) buffer, pH 8.0). DNA samples were sonicated with a probe sonicator (Bandelin Sonoplus, London, UK; UW 2070, Probe: SH70G) at 50% power for 10 s before they were heat-denatured at 96 °C for 5 min. Two pieces of Whatman 3 MM chromatography paper (GE Healthcare Life Sciences, Little Chalfont, Buckinghamshire, UK) and one piece of Amersham Hybond-N+ membrane (GE Healthcare Life Sciences) cut to fit the Minifold II Slot-Blot system (Schleicher & Schuell, London, UK) were pre-wet with TE buffer. This system was assembled according to the manufacturer’s instructions and connected to a vacuum water pump (600 mbar pressure). One μg of DNA was loaded into triplicate wells, allowed to dry and 100 μL of TE buffer was added to each well and left to dry again. The membrane was removed and placed in an oven at 80 °C for 30 min and then blocked using 5% skimmed milk (Marvel) dissolved in 0.02% Tween 20 in PBS (PBST) for 1 h at RT on a platform shaker. The membrane was incubated with HRP-MBP-Atl1 conjugate diluted 1:64,000 in 0.02% Tween 20 and 0.3% BSA in PBS (0.3 BPT), washed three times with 0.02% Tween 20 in PBS (PBST), placed in a clean dish, and wetted with Pierce enhanced chemiluminescence (ECL) Western blotting substrate (Thermo Fisher Scientific, Altrincham, Cheshire, UK). The membrane was then blotted, wrapped with cling wrap and exposed to Amersham Hyperfilm ECL (GE Healthcare Life Sciences, Little Chalfont, Buckinghamshire, UK) for between 30 s and up to one hour, according to the intensity of the bands on the developed film. The film was processed using a JP-33 Automatic X-ray Film Processor (JPI Healthcare Solutions, New York, NY, USA) and scanned (Scanjet 4850; Hewlett Packard, Manchester, UK). The intensity of the bands was quantified using ImageJ (version 1.53e). For each assay of human DNA, a standard curve was generated from the TMZ-treated series and the levels of *O*^6^-AlkG in the samples calculated from standard curves. The limit of detection (LOD) and limit of quantification (LOQ) was ~0.6 and 1.0 fmoles *O*^6^-AlkG/μg DNA, respectively, as calculated using the following formula: LOD = 3 (SD/S) and LOQ =10 (SD/S) where SD is the standard deviation of the blank and S is the slope of the calibration curve.

Optimisation of the ASB assay explored the type of membrane used, sample preparation method, the effect of different DNA concentrations, membrane washing method and membrane baking step. The solvent used in preparing the peroxidase-MBP-Atl1 conjugate and its optimum dilution were also investigated in attempts to increase the sensitivity of the assay. It is worth noting that initial assays using human DNA samples indicated that the signal for some were lower than the background signal for untreated CT-DNA. In attempts to find a DNA control with a lower background, Salmon testes, *Micrococcus luteus* and human placental DNA were assessed in the optimised ASB assay.

#### 2.4.1. Analysis of Membrane-Bound DNA by Propidium Iodide Staining

Following the ASB analysis, membranes were washed with 30 mL PBS for one hour, incubated with 30 mL of propidium iodide (PI) (5 μg/mL; Sigma Aldrich, Poole, UK), protected from light and then washed again with PBS for 30 min before being left to dry at room temperature for 20 min. Membranes were scanned (Typhoon 9200, GE Healthcare, Little Chalfont, Buckinghamshire, UK) at 560 nm and 250 V and the intensity of DNA bands was quantified using ImageJ software 1.8.0.

#### 2.4.2. MGMT-Treated DNA Sample Preparation

To 90 uL aliquots of DNA samples at 100 µg/mL in IBSA buffer (50 mM Tris pH 8.3, 3 mM DTT, 1 mM EDTA and 1 mg/mL BSA) was added 10 µL of His-MGMT (containing 70 pmoles by activity) or IBSA. Samples were incubated at 37 °C for 2 h, buffer I (IBSA without BSA) added and the samples vortex mixed, sonicated and heat denatured. DNA aliquots of 1 μg were analysed using the ASB as described above.

### 2.5. Culture and Treatment of MCF-10A Cells

MCF 10A cells were cultured in Dulbecco’s Modified Eagle’s Medium/Nutrient Mixture F-12 (DMEM/F12; Invitrogen, Inchinnan, Renfrewshire, Scotland), supplemented with 5% horse serum (Invitrogen), 20 ng/mL of epidermal growth factor (EGF; Peprotech, Thermo Fisher Scientific, Altrincham, Cheshire, UK), 0.5 mg/mL of hydrocortisone, 100 ng/mL of cholera toxin, 10 µg/mL of insulin and 1% penicillin-streptomycin mixture (Invitrogen) in an incubator supplied with 5% CO_2_, 3% O_2_ and set at 37 °C. Cells were treated with 500 µg TMZ for 72 h and DNA was extracted using the Blood and Cell Culture DNA Kit (Qiagen, Manchester, UK). It was then analysed using the ASB assay, together with untreated MCF 10A DNA and 1% DMSO-treated MCF 10A (DMSO-MCF 10A) cellular DNA as the negative controls.

## 3. Results

### 3.1. ASB Assay Detects a Range of Different O^6^-AlkGs in Alkylated CT DNA

**Figure 1:**
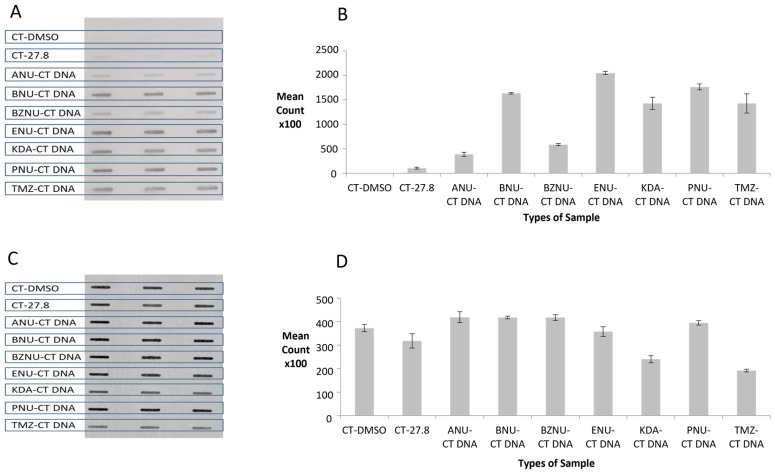
Binding of HRP-MBP-Atl1 to CT DNA treated with different alkylating agents. (**A**) ASB ECL images; (**B**) Atl1 binding quantitation; (**C**) PI staining images; (**D**) PI staining quantitation. Data represent mean ± SD of triplicate DNA samples. Abbreviations: CT-DMSO—calf thymus DNA incubated with DMSO; CT-27.8—calf thymus DNA incubated with temozolomide (103 μM) resulting in an *O*^6^-MeG level of 27.8 fmoles/µg DNA; ANU—allylnitrosourea; BNU—butylnitrosourea; BZNU —benzylnitrosourea; ENU—ethylnitrosourea; KDA—potassium diazoacetate; PNU—propylnitrosourea; TMZ—temozolomide.

In order to test the hypothesis that the ASB assay would be able to detect a wide range of *O*^6^-AlkGs in DNA, we analysed CT DNA treated with six different alkylating agents together with DMSO-only treated CT DNA as the negative control and TMZ-treated CT DNA as the positive control. The membrane image and the Image J-generated bar chart presented in Figure 1A and Figure 1B, respectively, showed strong signals compared to the negative control, demonstrating the ability of the ASB assay to detect a range of different *O*^6^-AlkGs.

The membrane was then stained with PI to assess the amounts of DNA binding to the membrane. Figure 1C,D show that, with the exception of KDA- and TMZ-treated DNA, broadly similar amounts of alkylated DNAs were bound.

### 3.2. Investigation of ISB Background Levels

**Figure 2:**
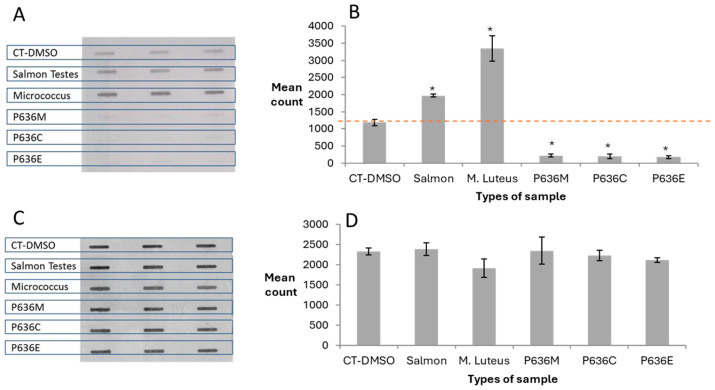
Binding of HRP-MBP-Atl1 to DNA samples not treated with alkylating agents. (**A**) ASB ECL images; (**B**) Atl1 binding quantitation; (**C**) PI staining images; (**D**) PI staining quantitation. Data represent mean ± SD of triplicate DNA samples. * Levels significantly different from CT-DMSO *p* < 0.05. Abbreviations: CT-DMSO—calf thymus DNA incubated with DMSO; P—placenta; C—cord region of placenta; M—middle region of the placenta; E—edge region of placenta. All placenta samples were from the same woman.

With some of the human DNA samples analysed initially, the ASB signals were lower than the background signal for negative control CT-DNA raising the possibility that *O*^6^-AlkGs were present in this control DNA. Three other DNA types not knowingly exposed to alkylating agents were therefore analysed by the ASB assay and a wide range of HRP-MBP-Atl1 binding levels were observed (Figure 2A,B). Salmon testis DNA displayed approx. 50% higher and *Micrococcus luteus* DNA was more than twice the levels seen with CT DNA. In contrast, the three placental DNA samples showed significantly lower levels compared to CT DNA. These differences were not due to different amounts of DNA as shown by the PI staining analysis (Figure 2C) which demonstrated similar levels of DNA binding to the membrane (Figure 2D).

### 3.3. HRP-MBP-Atl1 Binding to TMZ-CT DNA Is Reduced by Pretreatment with MGMT

**Figure 3:**
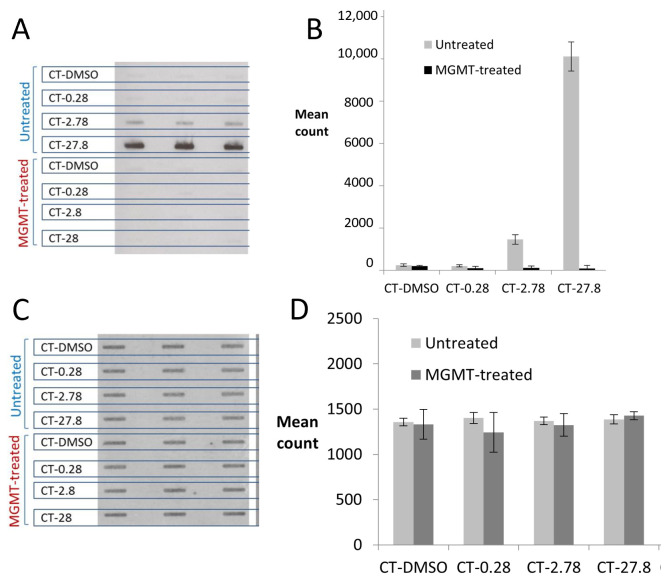
Effect of preincubation with MGMT on binding of HRP-MBP-Atl1 to TMZ-CT DNA. (**A**) ASB ECL images; (**B**) Atl1 binding quantitation; (**C**) PI staining images; (**D**) PI staining quantitation. Data represent mean ± SD of triplicate samples. DNA samples were untreated or MGMT-treated prior to processing for the ISB. Abbreviations: CT-DMSO—calf thymus DNA incubated with DMSO; CT-0.28, CT-2.78, CT-27.8—calf thymus DNA incubated with temozolomide (1.03–103 μM) resulting in *O*^6^-MeG levels of 0.28, 2.78 and 27.8 fmoles/µg DNA, respectively.

Aliquots of the TMZ-CT DNA samples described in Section 2.3 were incubated with MGMT as described in Section 2.4.2 and analysed using the ASB assay. The membrane images (Figure 3A) and ImageJ analyses (Figure 3B) showed that the HRP-MBP-Atl1 signals were almost completely ablated by preincubation with MGMT. PI staining of the membrane confirmed that the manipulations did not affect DNA binding (Figure 3C,D).

### 3.4. Establishing a Standard Curve Using TMZ-Treated DNA

**Figure 4:**
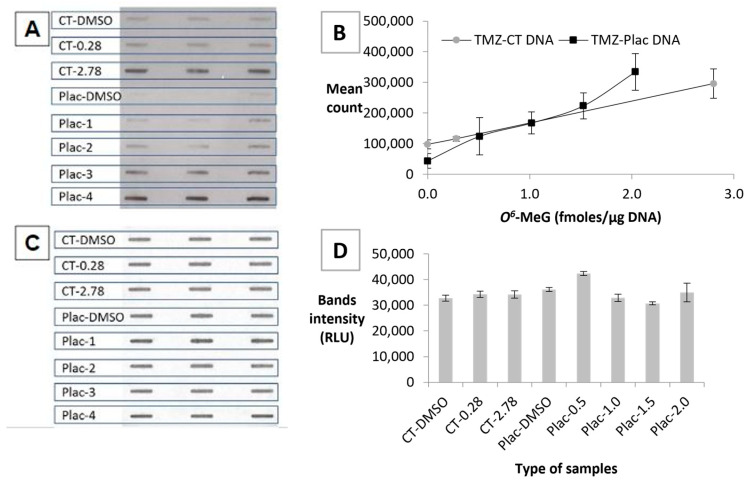
Binding of HRP-MBP-Atl1 to TMZ-CT DNA and TMZ-Plac DNA standards. (**A**) ASB ECL images; (**B**) *O^6^*-MeG standard curves; (**C**) PI staining images; (**D**) PI staining quantitation. Data represent mean ± SD of triplicate DNA samples. Abbreviations: CT-DMSO—calf thymus DNA incubated with DMSO; CT-0.28, CT-2.78—calf thymus DNA incubated with temozolomide (1.03–10.3 μM) resulting in *O*^6^-MeG levels of 0.28 and 2.78 fmoles/µg DNA, respectively; Plac-DMSO—placental DNA incubated with DMSO; Plac-0.5 (Plac-1), Plac-1.0 (Plac-2), Plac-1.5 (Plac-3) and Plac-2.0 (Plac-4)—placental DNA incubated with temozolomide (1.9–7.7 μM) resulting in *O*^6^-MeG levels of 0.5, 1.0, 1.5 and 2.0 fmoles/µg DNA, respectively.

Two sets of standards made by treating CT or human placental DNA with a range of concentrations of TMZ were then analysed using the ASB assay. The levels of O^6^-MeG in these samples had been determined using an MGMT-based competition assay. The ASB-ECL image is presented in Figure 4A and standard curves for both series of standards are plotted in Figure 4B. The limit of detection for TMZ-CT DNA and TMZ-Plac DNA was 0.61 and 0.55 fmoles/μg DNA, respectively.

To confirm that similar amounts of DNA had been bound to the membrane, it was stained with PI and the resulting image and Image J analysis are shown in Figure 4C and Figure 4D, respectively. The amount of DNA binding to the membrane has minimal effect on the different levels of *O*^6^-MeG detected in the samples. The lower background seen with the placental DNA sample makes this series the better option to be used in the ASB assay.

### 3.5. Analysis of Human Breast Tumour Cell Line DNA Samples

Cellular DNA extracted from MCF 10A cells treated with 500 µg TMZ (TMZ-MCF 10A) 1% DMSO (DMSO-MCF 10A) or untreated MCF 10A (MCF 10A) was then analysed using the ASB assay, using TMZ-Plac DNA as the standard curve. HRP-MBP-Atl1 binding was detected in all three samples and quantified using the standard curve. The highest level of binding was seen in TMZ-MCF 10A cells (equivalent to 1.34 fmoles/µg *O*^6^-MeG DNA), followed by DMSO-MCF 10A cells 0.46 fmoles/µg DNA and untreated MCF 10A cells (0.23 fmoles/µg DNA).

### 3.6. Analysis of Human DNA Samples

**Figure 5:**
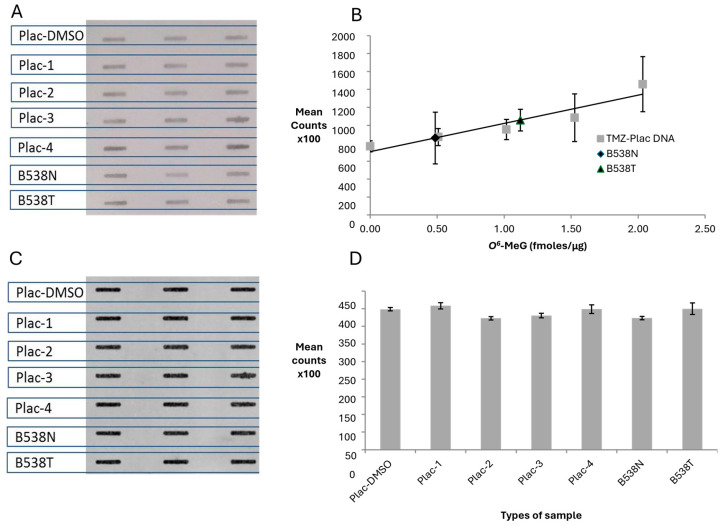
Binding of HRP-MBP-Atl1 to human breast tissue DNA and TMZ-treated placental DNA standards. (**A**) ASB ECL images; (**B**) HRP-MBP-Atl1 binding quantitation; (**C**) PI staining images; (**D**) PI staining quantitation. Data represent mean ± SD of triplicate samples. Abbreviations: Plac-DMSO—placental DNA incubated with DMSO; Plac-0.5 (Plac-1), Plac-1.0 (Plac-2), Plac-1.5 (Plac-3) and Plac-2.0 (Plac-4)—placental DNA incubated with temozolomide (1.9–7.7 μM) resulting in *O*^6^-MeG levels of 0.5, 1.0, 1.5 and 2.0 fmoles/µg DNA. B538 and B538T refer to normal and tumour samples, respectively, from the same patient.

Human breast DNA samples obtained from women with breast cancer were analysed by the ASB assay. Binding of HRP-MBP-Atl1 indicating the presence of *O*^6^-AlkG adducts in five tumour DNA samples (mean ± SD 1.24 ± 0.25; range 0.98–1.53 *O*^6^-meG equivalents/μg DNA) with trace levels detected in one other sample (Figure 5). DNA samples from normal breast tissue from two of these women were also analysed but only trace levels of adducts were detected. To confirm that similar amounts of DNA had been bound to the membrane, it was stained with PI and the resulting image and Image J analysis (Figure 5C and Figure 5D, respectively) show very similar levels of DNA binding to the membrane.

## 4. Discussion

In this study, we developed a new slot-blot assay to detect *O*^6^-AlkGs in human DNA using Atl1, a DNA-damage-sensing protein that recognizes *O*^6^-AlkGs [28]. This ASB assay detected a wide range of *O*^6^-AlkGs and MGMT was used to confirm that the ASB binds to *O*^6^-AlkGs. The assay was then applied to a limited series of human DNA samples with *O*^6^-AlkGs detected.

One of the original observations for the ATL protein of *Escherichia coli* (eATL) was that it could prevent the action of MGMT on *O*^6^-MeG in methylated DNA, suggesting strong binding to this lesion [30]. Further studies used electrophoretic mobility shift assays (EMSAs) to demonstrate that eATL and Atl1 bind to oligonucleotides containing a wide variety of single *O*^6^-AlkG residues [28,30]. Surface plasmon resonance (SPR) studies with several *O*^6^-AlkG-containing oligos showed similar binding (“on”) rates while dissociation (“off”) rates were slower for more complex alkyl groups such as benzyl or bromothenyl than for simple alkyl groups such as methyl or carboxymethyl. In contrast, in SPR, binding to control oligos was minimal and transient [28].

We first confirmed that the assay would be capable of binding to DNAs alkylated in vitro with a variety of direct acting alkylating agents (AA) and then bound to nylon membranes in a slot-blot apparatus. Instead of using anti-Atl1 antibodies and secondary antibodies coupled to HRP and then ECL substrates, we conjugated HRP directly to MBP-Atl1. The AA-treated DNAs were expected to contain methyl, ethyl, propyl, butyl, benzyl, allyl and carboxymethyl groups and binding to these was observed using HRP-MBP-Atl1, confirming essentially what had been seen with oligonucleotides using EMSA and SPR [28]. We then showed that, as expected, pretreatment of TMZ-methylated DNA with MGMT prior to membrane binding ablated the signal, suggesting that Atl1 was binding only to *O*^6^-MeG in this DNA and did not bind to other methylation products generated by TMZ such as N7-methylguanine or N3-methyladenine [31].

The sensitivity of such assays is determined by the signal-to-noise ratio and it had been assumed that any DNA not knowingly treated with an AA would provide a suitable background negative control. Although cooperative binding might occur during incubation of the membrane with the HRP-MBP-Atl1 conjugate, the SPR observations suggest that this would be reversed during the membrane washing steps. Certainly, the alkylated DNA samples consistently showed much higher levels of binding than the background CT DNA controls. However, pilot studies with human DNA samples showed Atl1 binding that was at a much lower level than the supposed untreated control CT DNA. This may be due to CT-DNA containing *O*^6^-AlkG adducts, as low levels of *O*^6^-MeG have previously been reported in untreated CT-DNA using an immunoslot-blot assay [32]. In attempts to identify a commercially available DNA that would serve as a negative control, we assessed salmon testis and *M.luteus* DNA. Surprisingly, the signals obtained with these were substantially higher than that from control CT DNA. Even in a cultured breast tumour cell line, the untreated or DMSO-treated control DNAs showed binding in the ASB assay. Since the lowest background was seen with placental DNA, this was used in subsequent assays as a background control. The nature of the background binding in other control DNA samples have not been pursued but endogenous DNA-methylating agents have been generated by nitrosation in *Escherichia coli* [8].

*O*^6^-AlkGs in human DNA have previously been quantified using methodology such as immunoslot-blots [ISB: 33] and mass-spectroscopic methods [22] to detect specific *O*^6^-AlkGs: detection limits for these assays are similar to those reported here with that for the *O*^6^-CMG ISB reported to be 0.4 fmole/μg DNA [33] and that for *O*^6^-meG by an UHPLC-HRMS/MS method [22] which detected 28.1 adducts per 10^8^ nucleotides which corresponds to ~0.9fmole *O*^6^-MeG/μg DNA. The main advantage of the ASB assay is that because Atl1 binds to such a wide range of *O*^6^-AlkGs, all such species would be quantified at the same time and so the assay could be considered to quantify the total potential genotoxicity burden from all *O*^6^-AlkGs. This ASB depends upon the ability of Atl1 to bind specifically to *O*^6^-AlkGs and so any *O*^6^-AlkGs that do not bind to Atl1 or bind with low affinity or are present in very low levels may not be detected. Hence, those *O*^6^-AlkGs that are efficiently repaired by MGMT will be less readily detected. Furthermore, a similar signal in the ASB from two different samples might have been generated by a widely different combination of *O*^6^-AlkGs of different biological potencies. However, as mentioned above, we have recently developed an assay that might be considered complementary to the ASB that is based on the ability of MGMT to capture alkyl groups onto the active site cysteine residue and the determination of the mass of the captured group by mass spectrometry [26]. Screening of DNA samples using the ASB assay could then lead to a subsequent focused analysis using the MS approach to identify which *O*^6^-AlkGs are present.

## 5. Conclusions

DNA adducts of the *O*^6^-AlkG type are pro-mutagenic and hence carcinogenic and exposure to *O*^6^-alkylators is ubiquitous. A screening method that is able to quantify these adducts in human DNA might therefore find widespread application. Towards this aim, we have developed a slot-blot-based assay to detect *O*^6^-AlkGs in human DNA samples using Atl1, a DNA-damage-sensing protein that binds to *O*^6^-AlkGs. This ASB assay detected a range of *O*^6^-AlkGs and MGMT pretreatment of the alkylated DNA samples has been used to confirm that the ASB detects *O*^6^-AlkGs. The assay was then applied to a series of human DNA samples with *O*^6^-AlkGs detected in most of them.

## Data Availability

The data presented in this study are available on request from the corresponding author.

## References

[1] Magee P.N., Barnes J.M. (1956). The production of malignant primary hepatic tumours in the rat by feeding dimethylnitrosamine. Br. J. Cancer.

[2] Druckrey H., Preussmann R., Ivankovic S., Schmähl D. (1967). Organotropic carcinogenic effects of 65 various N-nitroso- compounds on BD rats. Z. Krebsforsch..

[3] NTP (National Toxicology Program) (2021). Report on Carcinogens.

[4] Kobets T., Smith B.P.C., Williams G.M. (2022). Food-Borne Chemical Carcinogens and the Evidence for Human Cancer Risk. Foods.

[5] Schrenk D., Bignami M., Bodin L., Chipman J.K., Del Mazo J., Hogstrand C., Ron Hoogenboom L., Leblanc J.C., Nebbia C.S., EFSA Panel on Contaminants in the Food Chain (EFSA CONTAM Panel) (2023). Risk assessment of N-nitrosamines in food. EFSA J..

[6] Gushgari A.J., Halden R.U. (2018). Critical review of major sources of human exposure to N-nitrosamines. Chemosphere.

[7] Tricker A.R. (1997). N-nitroso compounds and man: Sources of exposure, endogenous formation and occurrence in body fluids. Eur. J. Cancer Prev..

[8] Taverna P., Sedgwick B. (1996). Generation of an Endogenous DNA-Methylating Agent by Nitrosation in Escherichia coli. J. Bacteriol..

[9] Hu C.W., Shih Y.M., Liu H.H., Chiang Y.C., Chen C.M., Chao M.R. (2016). Elevated urinary levels of carcinogenic N-nitrosamines in patients with urinary tract infections measured by isotope dilution online SPE LC-MS/MS. J. Hazard Mater..

[10] Rietjens I.M.C.M., Michael A., Bolt H.M., Siméon B., Andrea H., Nils H., Christine K., Angela M., Gloria P., Daniel R. (2022). The role of endogenous versus exogenous sources in the exposome of putative genotoxins and consequences for risk assessment. Arch. Toxicol..

[11] Li Y., Hecht S.S. (2022). Metabolic Activation and DNA Interactions of Carcinogenic N-Nitrosamines to Which Humans Are Commonly Exposed. Int. J. Mol. Sci..

[12] Drabløs F., Feyzi E., Aas P.A., Vaagbø C.B., Kavli B., Bratlie M.S., Peña-Diaz J., Otterlei M., Slupphaug G., Krokan H.E. (2004). Alkylation damage in DNA and RNA--repair mechanisms and medical significance. DNA Repair.

[13] Fahrer J., Christmann M. (2023). DNA Alkylation Damage by Nitrosamines and Relevant DNA Repair Pathways. Int. J. Mol. Sci..

[14] Beranek D.T. (1990). Distribution of methyl and ethyl adducts following alkylation with mono- functional alkylating agents. Mutat. Res..

[15] Shrivastav N., Li D., Essigmann J.M. (2010). Chemical biology of mutagenesis and DNA repair: Cellular responses to DNA alkylation. Carcinogenesis.

[16] Thomas A.D. (2020). Biological Basis for Threshold Responses to Methylating Agents. Chem. Res. Toxicol..

[17] Wang P., Wang Y. (2018). Cytotoxic and mutagenic properties of *O*^6^-alkyl-2′-deoxyguanosine lesions in Escherichia coli cells. J. Biol. Chem..

[18] Knijnenburg T.A., Wang L., Zimmermann M.T., Chambwe N., Gao G.F., Cherniack A.D., Fan H., Shen H., Way G.P., Greene C.S. (2018). Genomic and Molecular Landscape of DNA Damage Repair Deficiency across the Cancer Genome Atlas. Cell Rep..

[19] Gurjao C., Zhong R., Haruki K., Li Y.Y., Spurr L.F., Lee-Six H., Reardon B., Ugai T., Zhang X., Cherniack A.D. (2021). Discovery and Features of an Alkylating Signature in Colorectal Cancer. Cancer Discov..

[20] Hall C.N., Badawi A.F., O’Connor P.J., Saffhill R. (1991). The detection of alkylation damage in the DNA of human gastrointestinal tissues. Br. J. Cancer.

[21] Wilson V.L., Weston A., Manchester D.K., Trivers G.E., Roberts D.W., Kadlubar F.F. (1989). Alkyl and aryl carcinogen adducts detected in human peripheral lung. Carcinogenesis.

[22] Hemeryck L.Y., Decloedt A.I., Vanden Bussche J., Geboes K.P., Vanhaecke L. (2015). High resolution mass spectrometry-based profiling of diet-related deoxyribonucleic acid adducts. Anal. Chim. Acta.

[23] Tessmer I., Margison G.P. (2024). The DNA Alkyltransferase Family of DNA Repair Proteins: Common Mechanisms, Diverse Functions. Int. J. Mol. Sci..

[24] Fang Q. (2024). The Versatile Attributes of MGMT: Its Repair Mechanism, Crosstalk with Other DNA Repair Pathways, and Its Role in Cancer. Cancers.

[25] Kaina B., Christmann M., Naumann S., Roos W.P. (2007). MGMT: Key node in the battle against genotoxicity, carcinogenicity and apoptosis induced by alkylating agents. DNA Repair.

[26] Abdelhady R., Senthong P., Eyers C.E., Reamtong O., Cowley E., Cannizzaro L., Stimpson J., Cain K., Wilkinson O.J., Williams N.H. (2023). Mass Spectrometric Analysis of the Active Site Tryptic Peptide of Recombinant *O*^6^-Methylguanine-DNA Methyltransferase Following Incubation with Human Colorectal DNA Reveals the Presence of an *O*^6^-Alkylguanine Adductome. Chem. Res. Toxicol..

[27] Pearson S.J., Wharton S., Watson A.J., Begum G., Butt A., Glynn N., Williams D.M., Shibata T., Santibáñez-Koref M.F., Margison G.P. (2006). A novel DNA damage recognition protein in Schizosaccharomyces pombe. Nucleic Acids Res..

[28] Latypov V.F., Tubbs J.L., Watson A.J., Marriott A.S., McGown G., Thorncroft M., Wilkinson O.J., Senthong P., Butt A., Arvai A.S. (2012). Atl1 regulates choice between global genome and transcription-coupled repair of O6-alkylguanines. Mol. Cell..

[29] Watson A.J., Middleton M.R., McGown G., Thorncroft M., Ranson M., Hersey P., McArthur G., Davis I.D., Thomson D., Beith J. (2009). *O*^6^-methylguanine-DNA methyltransferase depletion and DNA damage in patients with melanoma treated with temozolomide alone or with lomeguatrib. Br. J. Cancer.

[30] Pearson S.J., Ferguson J., Santibanez-Koref M., Margison G.P. (2005). Inhibition of *O*^6^-methylguanine-DNA methyltransferase by an alkyltransferase-like protein from Escherichia coli. Nucleic Acids Res..

[31] Sobol R.W., Schwab M. (2008). Temozolomide. Encyclopedia of Cancer.

[32] Göder A., Nagel G., Kraus A., Dörsam B., Seiwert N., Kaina B., Fahrer J. (2015). Lipoic acid inhibits the DNA repair protein *O*^6^-methylguanine-DNA methyltransferase (MGMT) and triggers its depletion in colorectal cancer cells with concomitant autophagy induction. Carcinogenesis.

[33] Cupid B.C., Zeng Z., Singh R., Shuker D.E.G. (2004). Detection of *O*^6^-carboxymethyl-2′-deoxyguanosine in DNA following reaction of nitric oxide with glycine and in human blood DNA using a quantitative immunoslot blot assay. Chem. Res. Toxicol..

